# An Essential Requirement for *Fgf10* in Pinna Extension Sheds Light on Auricle Defects in LADD Syndrome

**DOI:** 10.3389/fcell.2020.609643

**Published:** 2020-12-10

**Authors:** Yang Zhang, Juan M. Fons, Mohammad K. Hajihosseini, Tianyu Zhang, Abigail S. Tucker

**Affiliations:** ^1^Centre for Craniofacial and Regenerative Biology, King’s College London, London, United Kingdom; ^2^Ear Nasal and Throat (ENT) Institute, Eye and Ear Nose and Throat Hospital, Fudan University, Shanghai, China; ^3^School of Biological Sciences, University of East Anglia, Norwich, United Kingdom; ^4^Department of Facial Plastic and Reconstructive Surgery, Eye & Ear Nose and Throat Hospital, Fudan University, Shanghai, China

**Keywords:** ear development, auricle, congenital birth defect, muscle, fibroblast growth factor, microtia

## Abstract

The pinna (or auricle) is part of the external ear, acting to capture and funnel sound toward the middle ear. The pinna is defective in a number of craniofacial syndromes, including Lacrimo-auriculo-dento-digital (LADD) syndrome, which is caused by mutations in *FGF10* or its receptor *FGFR2b*. Here we study pinna defects in the *Fgf10* knockout mouse. We show that Fgf10 is expressed in both the muscles and forming cartilage of the developing external ear, with loss of signaling leading to a failure in the normal extension of the pinna over the ear canal. Conditional knockout of *Fgf10* in the neural crest fails to recapitulate this phenotype, suggesting that the defect is due to loss of *Fgf10* from the muscles, or that this source of *Fgf10* can compensate for loss in the forming cartilage. The defect in the *Fgf10* null mouse is driven by a reduction in proliferation, rather than an increase in cell death, which can be partially phenocopied by inhibiting cell proliferation in explant culture. Overall, we highlight the mechanisms that could lead to the phenotype observed in LADD syndrome patients and potentially explain the formation of similar low-set and cup shaped ears observed in other syndromes.

## Introduction

Microtia is a common congenital birth defect, wherein the external pinna (or auricle) is small and/or abnormally formed. It is observed with an incidence of 0.83–17.4 per 10,000 births, depending on geographical location (Luquetti et al., 2012). 12.6% of microtia patients have an underlying craniofacial syndrome (Cabrejo et al., 2019). Microtia is often associated with defects in the middle ear, which together can lead to conductive hearing loss (Cox et al., 2014). During the process of hearing, the pinna acts as a funnel, and can also provide information regarding the location of sound (Hofman et al., 1998). In addition, in human beings, the pinna plays a major role in our appearance, and as such abnormally shaped pinnas impact on daily life. Microtia is treated by ear reconstruction surgery or the use of ear molds in infants to correct the shape of the pinna (Chan et al., 2019). Ear surgery is challenging because the pinna’s appearance varies considerably between patients.

The pinna is part of the outer ear, which also contains the ear canal and tympanic membrane. A true pinna is only found in therian mammals (marsupials and eutherians), and is thought to have developed after their evolutionary divergence from the egg laying monotremes (Mozaffari et al., 2020). The pinna derives largely from the second pharyngeal arch in mouse, while it is thought to develop from the first and second pharyngeal arch in humans, with the tragus being first arch derived (Minoux et al., 2013). The mouse pinna develops slightly differently from the human pinna. In the mouse, the pinna initiates at Embryonic day (E)11.5. As the mouse pinna grows it bends toward the rostral part of the head, extending a flap over the ear canal to cover the ear canal completely by E18.5 (Cox et al., 2014). The pinna then fuses to the side of the head and remains, encasing the ear canal until postnatal stages. At 3–5 days postnatally, the pinna flap detaches from the head, lifts up and flips back to reach its adult position (Anthwal and Thompson, 2016). The pinna continues to grow postnatally reaching its adult shape and size at around postnatal day 14. In humans, the main part of the pinna remains posterior to the ear canal during development, forming a complex folded structure (Cox et al., 2014), with the majority of growth completed by 9 years old. However, pinnae continue to grow and male pinnae are bigger than female (Sforza et al., 2009).

Several craniofacial syndromes are associated with pinna defects, such as Lacrimo-auriculo-dento-digital syndrome (LADD) (MIN14970), Branchio-oto-renal (BOR) syndrome and 22q11.2 deletion syndrome amongst others (Thompson et al., 1985; Oskarsdottir et al., 2008; Galliani et al., 2012). LADD syndrome is an autosomal-dominant multiple congenital anomaly disorder characterized by defects in lacrimal and salivary glands, the dentition, digits and ear (Milunsky et al., 2006). The main malformation of the pinna is the presence of a cup-shaped ear which is often low-set (Inan et al., 2006). LADD syndrome has been shown to be caused by defects in the FGF (fibroblast growth factor) signaling pathway, with mutations in *FGF10* and its receptor *FGFR2b*, both leading to the same phenotype (Milunsky et al., 2006; Rohmann et al., 2006). In mouse development, *Fgf10* is expressed in the glands, limb and in the forming pinna (El Agha et al., 2012; Teshima et al., 2016). Knockout of *Fgf10* in the mouse, leads to defects in glands and teeth (Ohuchi et al., 2000), although the pinna has been suggested to develop as normal (Prochazkova et al., 2018).

Fgf10 is a secreted protein composed of 250 amino acids usually acting in a paracrine manner (Yamasaki et al., 1996). It belongs to the Fgf7/10/22 subfamily of Fibroblast growth factors (Itoh and Ornitz, 2008). *Fgf10* plays a role in mesenchymal to epithelial interaction, important in many developing tissues and organs (Itoh, 2016). The *Fgf10* null mutant mouse dies at birth due to the absence of lungs, while conditional loss of *Fgf10* in the neural crest, mirrors a subset of the phenotype and leads to a similar death at birth due to the presence of a cleft palate (Teshima et al., 2016). In addition to lung defects, mutations in *Fgf10* cause limb aplasia due to a deregulation of the apical ectodermal ridge (AER) (Sekine et al., 1999). Mesenchymal *Fgf10* in the progress zone signals to the AER through *Fgfr2b* to upregulate *Wnt3a-Fgf8* axis that then feeds back to regulate *Fgf10* expression (Jin et al., 2018). In the salivary gland, loss of *Fgf10* leads to an arrest of gland development at the placode stage, with downregulation of Sox9 expression in the distal epithelial compartment (Chatzeli et al., 2017).

FGF signaling in embryogenesis is considered to be transduced by three major pathways, PLC γ, PI3Kinase/PKB and RAS/ERK1/2 [MAP (mitogen-activated protein) Kinase] (Bottcher and Niehrs, 2005). ERK1/2 has been shown to relay signaling from FGF receptors in early fish, frog, chick and mouse embryos (Christen and Slack, 1999; Corson et al., 2003; Lunn et al., 2007). In the lung, mesenchymal Fgf10 signals to the epithelium through Fgfr2b to promote proliferation and differentiation by activating the MAP kinase signaling pathway (Yin and Ornitz, 2020). MAPK signaling consists of a series of phosphorylation cascades involving 3 kinases, RAF, MEK 1/2 and MAP (ERK1/2) kinases. After nuclear translocation, p-ERK1/2 activates gene transcription of the PEA3 sub-family (PEA3/ETV4, ERM/ETV5 and ER81/ETV1), which have been utilized as readouts of FGF activity (Lunn et al., 2007). Mutations in *Erk1/2* mimic the defects observed in patients with *22q11.2* deletion syndrome, one of the craniofacial syndromes associate with microtia (Newbern et al., 2008). Defects in FGF signaling, acting via the RAS/ERK pathway, may, therefore, underlie the ear phenotype in both LADD syndrome and 22q11.2DS.

In this study we have investigated pinna development in *Fgf10* knockout mice in order to understand potential mechanisms involved in LADD syndrome. We highlight the source of Fgf10, the tissues that respond to Fgf10 signaling in the ear, and the cell processes affected. The *Fgf10* knockout mouse can therefore be used as a model to study the mechanisms underlying the pinna defect in LADD syndrome patients, and other syndromes with low set, cup shaped ears.

## Materials and Methods

### Animals

*Fgf10*^+/–^ mouse were intercrossed to generate *Fgf10* null embryos. *Fgf10*^+/–^ and *Fgf10*^+/+^ mice were both used as a control group for *Fgf10*^–/–^ as the heterozygous mice have normal pinnae. The reduction of *Fgf10* in the heterozygous mice leads to defects in gland development but does not affect breeding (May et al., 2015). *Fgf10^*fl/fl*^* females (*Fgf10A02 tmc1c*) were crossed to *Wnt1cre; Fgf10^*fl/*+^* to generate *Wnt1cre; Fgf10^*fl/fl*^* and *Fgf10^*fl/fl*^* used as controls (Teshima et al., 2016). To confirm the neural crest origin of the mesenchyme of the pinna, *Wnt1cre* mice were mated to *R26RtdTom* reporter mice. The matings were set up in the evening and the day of the vaginal plug observed was marked as E0.5. Pregnant females were culled with Schedule I culling methods at E14.5, E15.5, and E18.5. After culling, all embryos were photographed on a Leica dissecting microscope.

### Histology

After collection embryos were fixed in 4% paraformaldehyde (PFA) and then dehydrated in gradually increasing Ethanol concentrations (30, 50, 70, 90, 95, and 100% 1 h per step). Embryos were cleared in xylene and embedded in paraffin. Sections were cut at 8μm and stained with Alcian Blue, Sirius Red and Ehrlich’s Haematoxylin.

### LacZ Staining

Heads from *Fgf10^*nlacZ/*+^* mice (Kelly et al., 2001), were fixed in 4% PFA for 20 min, *N* = 3. To stain heads were washed twice for 20 min in PBS with 2 mM MgCl_2_. Heads were then incubated for 15 min in a solution containing 1 mM MgCl_2_, 0.2% NP-40 and 0.02% deoxycholic acid diluted in PBS (Solution B), and then stained with Solution C made with 5 mM K_3_Fe(CN)_6_, 5 mM K_4_Fe(CN)_6_ and 1 mg/ml x-gal diluted in Solution B. Staining was performed at 37^*o*^C for 4 h. Using the same protocol, cryosections on slides were additionally stained at 37^*o*^C for 4 days to increase the intensity of the signal, washed in PBS and re-fixed.

### Immunofluorescence and *in situ* Hybridization

Primary antibodies used include anti-BrdU (ab6326, abcam), anti-Caspase 3 (9661s, Cell Signaling Technology), anti-12-101 (AB_531892, DSHB), and anti-SOX9 (AB5535, Millipore), β-gal (ab9361, abcam), anti-P-p44/42 MAPK (9101s, Cell signaling) and anti-RFP (red fluorescent protein) (Chromotek 5f8-100). For Caspase 3, the signal was amplified with a TSA kit (PerkinElmer). For BrdU, 2M HCl at 37^*o*^C was used before addition of the primary antibody. *In situ* hybridization was performed as previously described (Fons Romero et al., 2017). Immunolabelings and *in situ* hybridization reactions were repeated at least 3 times to confirm the expression patterns.

### Rolling Explant Culture

In order to culture the whole pinna we used a novel rotational tissue culture system. CD1 pregnant females were sacrificed at E12.5. After halving the heads and removing the brain, embryos were place in KO-DMEM (A12861-01) medium in Falcon tubes. Proliferation inhibitor (Aphidicolin, sc-201535)(2 μg/ml) disolved in DMSO was applied to one group (*N* = 6) and DMSO (carrier) as a control to the other group (*N* = 16). Tubes were incubated at 37°C with 95% O_2_ and 5%CO_2_ gas (Carbogen) and rotated at 25 rpm to improve circulation for 2 days.

### Proliferation Analysis

For the *Fgf10* embryos at E14.5 and E15.5, pregnant females were IP injected with BrdU (30 mg/kg) 1 h before collection. Embryos were sectioned and immunostained for BrdU by IF and imaged. Defined groups of cells within the pinna were demarcated using a standardized method (see Supplementary Figure 1 for method used to define a standardized area for counting). The total number of nuclei that were BrdU positive within the defined region was counted on up to 10 continuous sections for each embryo using ImageJ. Counts for each embryo were then averaged and the average number of proliferating cells compared across littermates (*N* = 3–4 embryos per group).

### Statistics

A Student’s two tailed unpaired *t*-test was used for the proliferation comparison. Fisher exact probability was performed for comparisons. A significant difference was taken as *P* < 0.05 (^∗^).

## Results

### *Fgf10* Is Expressed in the Developing Muscle and Cartilaginous Condensations of the Pinna

**FIGURE 1:**
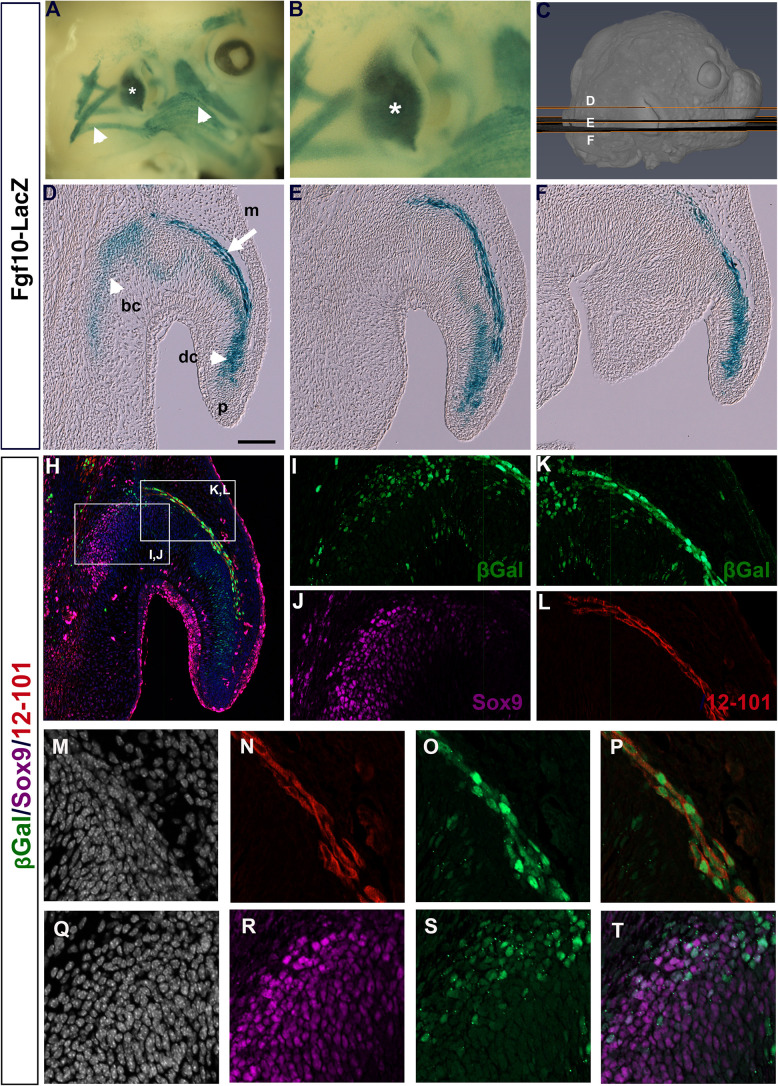
Fgf10 is expressed in auricular muscle and cartilage. **(A,B)** Whole mount βGal staining of *Fgf10*-LacZ reporter mouse at E14.5. Fgf10 is expressed in the pinna (asterisk) and the surrounding muscles (arrowheads). **(C)** μCT 3D reconstruction of a E14.5 embryo showing the level and plane of the respective sections. **(D–F)** Wax sections stained for βGal. Posterior is up, anterior is down. Fgf10 is expressed in the basal (bc) and distal cartilage (dc) and in the pinna muscle (m). Pinna tip = (p). **(H)** Triple IF for βGal (green), Sox9 (magenta) and 12–101 (red). **(I–L)** Higher magnification of boxed regions in **(H)**. **(I,J)** Fgf10 expression colocalizes with the cartilage marker Sox9 in the basal cartilage. **(K,L)** Fgf10 expression colocalizes with the muscle marker 12–101 in the pinna muscle. **(M–T)** Close up of the developing pinna muscle **(M–P)** and basal cartilage **(Q–T)**. **(M,Q)** DAPI showing nuclei in selected region. **(N)** 12–101 9 (red). **(O)** βGal (green). **(P)** Overlap between 12–101 and βGal. **(R)** Sox9 (magenta). **(S)** βGal (green). **(T)** Partial overlap between Sox9 and βGal. Scale bar in *D* = 100μm.

Previously, an *Fgf10icre/Tom* mouse has been used to show widespread expression of Fgf10 in the developing pinna at E18.5, traced from E15.5 (El Agha et al., 2012). To get a more detailed understanding of the expression of Fgf10 during pinna development we used *Fgf10LacZ* reporter mice, where LacZ is under the control of *Fgf10* regulatory sequences without disrupting Fgf10 function (Kelly et al., 2001). Fgf10 was expressed at high levels in the forming pinna at E (embryonic day) 14.5 (Figures 1A,B, asterisk), in addition to the surrounding craniofacial muscles (Figure 1A arrowheads). In section, at different planes through the pinna (see Figure 1C), a band of positive expression was evident following the curve of the pinna (Figures 1D–F, arrow in Figure 1D). In addition, Fgf10 was expressed in the developing cartilage extending from the base of the pinna (basal cartilage, bc) to the tip of the pinna (distal cartilage, dc) (Figures 1D–F, arrowhead in Figure 1D).

In order to confirm the expression of Fgf10 in the two different regions we compared the expression of LacZ with markers for early cartilage (Sox9) and differentiated muscle (12–101) (Kintner and Brockes, 1984; Lefebvre et al., 2019). Overlap of Sox9 and LacZ was found at the base of the pinna (Figures 1H–J,Q–T), confirming that some cartilage cells expressed *Fgf10*. Interestingly, we did not detect Sox9 expression in the cell condensates in the distal cartilage of the pinna (dc) at this stage of development, although Sox9 was upregulated in this region at later stages (data not shown), suggesting that Fgf10 predated Sox9 expression in this region. A convincing overlap was evident between 12–101 and LacZ, highlighting the expression of Fgf10 in the pinna muscle (Figures 1H,K,L,M–P). Fgf10 is therefore expressed in two populations in the developing pinna, the mesodermally-derived muscles of the growing pinna, and the neural crest-derived cartilage.

### Lack of *Fgf10* Results in a Shorter Pinna but Without Loss of Sox9 and 12–101

**FIGURE 2:**
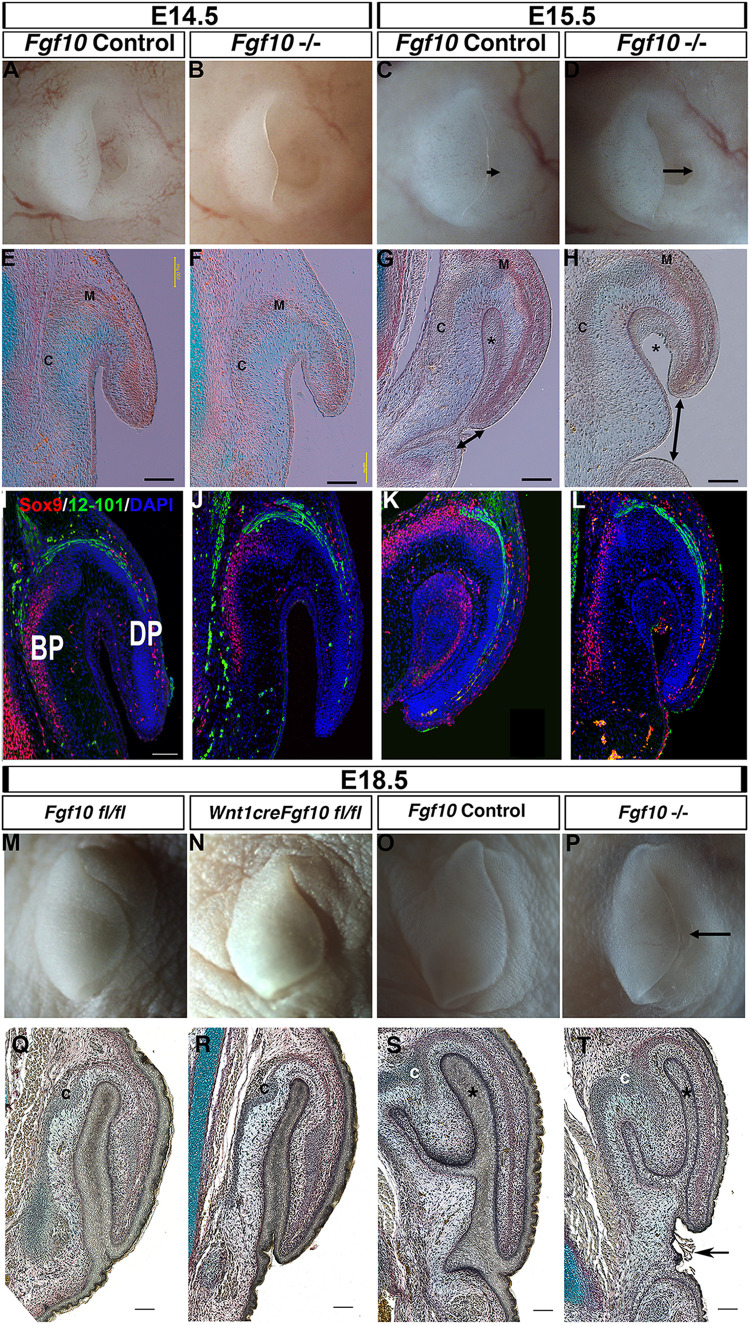
Fgf10 is required for pinna elongation. **(A–D)** whole mount pinnae at E14.5 **(A,B)** and E15.5 **(C,D)** of a *Fgf10* null mutant **(B,D)** and control littermate **(A,C)**. The phenotype start to be visible at E15.5 with a defect in the anterior elongation of the pinna (**C,D** arrow). **(E–H)** Trichrome staining. The defect in elongation is clear at E15.5 (**G,H** double arrow head). **(I–L)** Double IF for Sox9 and 12–101. Both markers are expressed in comparable domains to the wildtype. **(M)** *Fgf10* fl/fl control at E18.5. **(N)** Wnt1cre/+ *Fgf10* fl/fl at E18.5. **(O)** *Fgf10* control E18.5. **(P)** *Fgf10* null mutant at E18.5. **(Q–T)** Trichrome staining. Ablation of *Fgf10* in the neural crest cells does not phenocopy the elongation defects in the *Fgf10* null mutant (**P,T** arrow). (BP) basal pinna (DP) distal pinna. Asterisk labels the inner epithelial layer of the pinna that fuses with the adjacent head epithelium. (C) Forming cartilage. (M) forming muscle. Scale bar in **(E–H,I,Q–T)** = 100 μm. Same scale in **(J–L)**.

Previous analysis of the pinna in *Fgf10* knockout mice has not described a defect, and in keeping with this the pinna of *Fgf10* null mutants appeared normal at E14.5 (Figures 2A,B,E,F) (*N* = 5). *Fgf10*, therefore, does not appear to be involved in early initiation of the ear in mice. A phenotype, however, was evident from E15.5 (Figures 2C,D,G,H). By E15.5 in controls, the pinna had extended forward over the ear canal toward the prospective tragus, so that the opening was no longer evident (Figure 2C) (*N* = 6). In contrast, in the mutant the pinna did not extend leaving the ear canal exposed (Figures 2D,G,H, double arrow head) (*N* = 4). In section, the pinna was fused to the rest of the head in the controls, however, the mutant pinna remained detached from the head (compare Asterisk in Figures 2G,H) and far from the forming ear canal (arrowheads Figures 2G,H). The histology at all stages analyzed indicated formation of muscle and cartilage, which was confirmed by expression of the cartilage marker Sox9 and terminally differentiated muscle marker 12–101 (Figures 2I–L). Expression of muscle and cartilage markers was therefore unaffected by loss of *Fgf10*. In addition to its expression in cartilage, Sox9 was also expressed in the fused epithelium of the inner pinna at E15.5, while expression was not observed in this region prior to fusion (at E14.5) or in the unfused mutant at E15.5 (Figures 2I,K,L). Sox9 may therefore play an additional role in fusion of the pinna epithelium. By E18.5, the pinna was observed fused to the head in both mutant and wild-type littermates, however, the ear canal remained exposed and the pinna had failed to meet the future tragus in the mutants (Figures 2O,P,S,T) (*N* = 1).

In order to distinguish the role of mesodermal *Fgf10* from the neural crest derived *Fgf10* (Figure 1) we used a conditional approach to ablate *Fgf10* specifically in the cranial neural crest using Wnt1cre (Figures 2M,N,Q,R) (Teshima et al., 2016). The cartilage of the pinna is formed from neural crest derived mesenchyme, while the muscles are non-neural crest derivatives (Supplementary Figure 2). Agreeing with the previously published phenotype, the conditional mutants had cleft palates, confirming that that *Fgf10* had been deleted in the cranial neural crest (Supplementary Figure 3). In this conditional mutant, the pinna appeared identical to that of control littermates, in contrast with the *Fgf10* null mutants at this timepoint (Figures 2Q–T). Loss of *Fgf10* specifically in the neural crest population, therefore, had limited effect on pinna development (*N* = 2). This suggests that mesodermal *Fgf10* is the main source of Fgf10 for pinna elongation, or that in the absence of a source from the neural crest, mesodermal *Fgf10* can compensate for any loss.

### Pinna Extension Defect Is Caused by Reduced Cell Proliferation, Rather Than Cell Death

**FIGURE 3:**
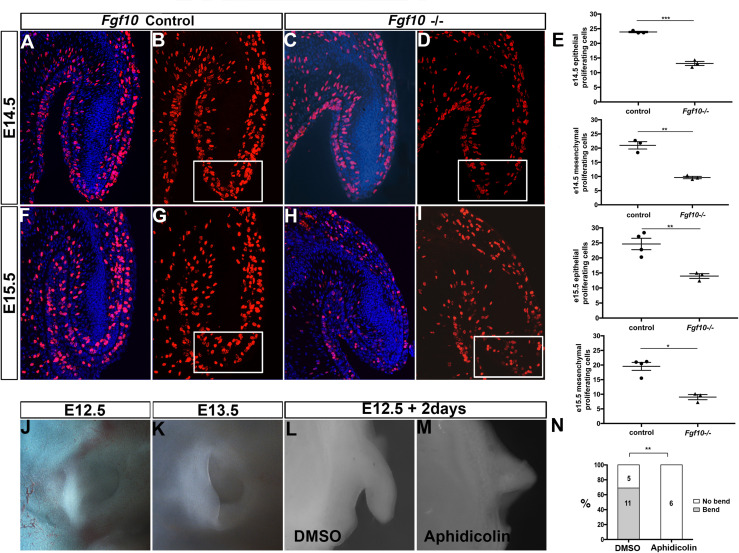
Proliferation is required for the bending and elongation of the pinna. BrdU IF at E14.5 **(A–D)** and at E15.5 **(F–I)** in control littermates **(A,B,F,G)** and *Fgf10* null mutant **(C,D,H,I)**. There is a reduction in BrdU positive cells at the tip of the pinna (box in **D,I**) in the mutant. **(E)** Quantification of the percentage of BrdU positive cells within the mesenchymal and epithelial at the tip of the pinna in the control and in the mutant at E14.5 and E15.5. E14.5 tip epithelium *P* = 0.0001. E14.5 tip mesenchyme *P* = 0.0011. E15.5 tip epithelium *P* = 0.0061. E15.5 tip mesenchyme *P* = 0.019. **(J,K)** Control embryonic pinnae at E12.5 and at E13.5. The pinna bends and elongates anteriorly. **(L,M)** Cultured whole pinnae in the presence **(M)** or absence **(L)** of the proliferation inhibitor Aphidicolin. After 2 days in culture, the pinna bends and elongates as observed during embryonic development (compared to **J,K**). Inhibition of proliferation abolishes both bending and elongation **(M)**. **(N)** Quantification of the growth and bending observed in cultured pinnae. *P* = 0.002. *P* < 0.05 (*), *P* < 0.01 (**), *P* < 0.001 (***).

FGFs are known to have a role in controlling both proliferation and apoptosis (Prochazkova et al., 2018). We therefore investigated whether proliferation and cell death were altered in the *Fgf10* null mutants using BrdU and activated Caspase 3. Since the phenotype started to be evident at E15.5, we analyzed proliferation at two stages, E14.5 and E15.5. As expected from a growing structure, high levels of cell proliferation were present in the epithelium and adjacent mesenchyme in control embryos at E14.5 and E15.5 (Figures 3A,B,F,G). In contrast, in the mutant embryos a reduction in proliferation was evident. To quantify this change, cells were counted in defined regions at the tip (boxes in Figures 3B,D,G,I), and at the sides (boxes in Supplementary Figure 4A) of the pinna (*N* = 3–4 embryos per group). Proliferation of both epithelial and mesenchymal cells was significantly reduced compared to the controls at both stages analyzed in the epithelium and mesenchyme, suggesting that the proliferation defect at E14.5 might drive the phenotype at E15.5 (Figure 3E and Supplementary Figure 4B). The most significant differences were evident in the extending tip, and outer pinna mesenchyme, but reduced proliferation was also evident in other regions of the pinna, suggesting a widespread reduction in proliferation in the external ear (Supplementary Figure 4B). To investigate whether the failure to extend the pinna was also due to increased programmed cell death, we followed activated Caspase 3 expression. No positive cells were identified in the developing pinna in mutants or littermates controls at E14.5 and E15.5 (Supplementary Figure 5) (*N* = 3). As a positive control, apoptotic cells were observed in the ear canal during normal development at E15.5 (Supplementary Figure 5F arrow), agreeing with the literature (Nishizaki et al., 1998; Fons et al., 2020). A reduction of proliferation, rather than an upregulation of cell death, therefore, appeared to underlie the defect.

In order to understand the role of proliferation in pinna development we then turned to a rolling culture technique to be able to perturb this process (Figures 3J–M). Wild type embryonic heads were divided down the midline and separated into two groups for culture. One group of half heads were cultured in the presence of the proliferation inhibitor Aphidicolin (2 μg/ml), while the other half were cultured in control medium. Use of Aphidicolin at this concentration has previously been shown to lead to a global inhibition of proliferation in explant culture (Yamada et al., 2019). Embryos older than E12.5 failed to develop well in rolling culture, presumably due to the larger size of the heads impacting on diffusion of nutrients, while in Trowel culture on membranes the pinna flattened onto the head and failed to extend. We therefore concentrated on E12.5. At E12.5 the pinna is an outgrowth that extends out perpendicular to the head. By E13.5 the pinna bends anteriorly and starts to elongate toward the prospective tragus (Figures 3J,K). After 2 days, we checked the growth of the pinnae. In the controls, 11 (out of 16) pinnae grew and folded over mimicking the normal process (Figures 3L,N). In contrast, none of the inhibitor-treated pinna grew or bent, phenocopying the failed extension of the *Fgf10* null mutant pinnae (Figures 3M,N, *P* = 0.002). This experiment highlights the importance of cell proliferation for the bending and the elongation of the pinna during development.

### Fgf10 Regulates the MAP Kinase Pathway During Pinna Elongation

**FIGURE 4:**
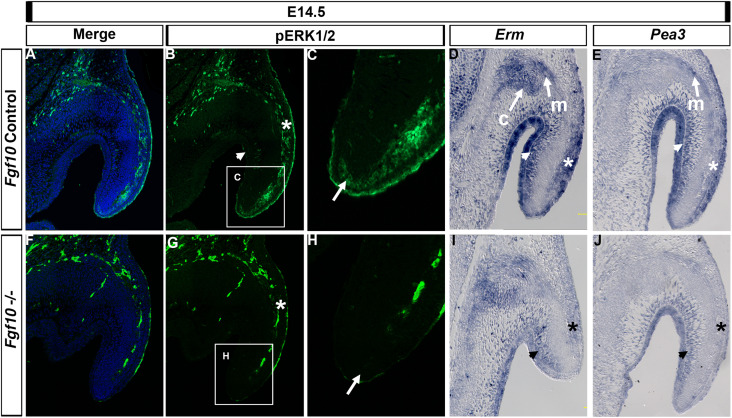
ERK1/2 is transducing Fgf10 signaling in the elongating pinna. IF for p-ERK1/2 at E14.5 in control littermates **(A–C)** and *Fgf10* null mutant **(F–H)**. **(C,H)** High magnification of the tip of the pinna in (box in **B,G**). ERK1/2 are phosphorylated in the outer mesenchymal and epithelium (**B,G**, asterisk) and at the tip (**C,H**, arrow) with an absence in the mutant. ISH for *Erm* **(D,I)** or *Pea3* **(E,J)**. *Erm* is expressed in the auricular muscle **(m)** and cartilage **(c)** and in the inner (**D**, arrow head) and outer (**D**, asterisk) mesenchyme and epithelium with a reduction in expression in the mutant **(I)**. *Pea3* is expressed in the auricular muscle **(m)** and inner (**E**, arrow head) and outer (**E**, asterisk) mesenchyme and epithelium with a reduction in expression in the mutant **(J).** c, cartilage; m, muscle.

FGF signaling regulates a number of pathways downstream of its receptor, of which the MAP kinase (RAS/RAF/ERK1/2) pathway is important in a number of developing organs, including the pinna (Newbern et al., 2008). We therefore investigated aspects of the RAS/ERK1/2 pathway. In control embryos at E14.5, phospho-ERK (p-ERK) was present in the epithelium and mesenchyme on the outer side of the pinna (Figures 4A–C, asterisk in B), extending down the pinna to the tip (Figure 4C, arrow). In contrast, expression was absent from the epithelial and mesenchymal cells on the inner side of the pinna, and from the Fgf10 expressing cartilage condensations in the middle of the pinna (Figure 4B, arrowhead). In the absence of *Fgf10*, p-ERK was reduced in the outer mesenchyme (Figures 4F,G, asterisk) with patchy expression in the surface epithelium and no detection of p-ERK at the tip (Figure 4H, arrow), highlighting the relevance of this region to the failure to extend the pinna. Blood vessels show up as autofluorescence in both cases and highlight the vasculature running down the outer side of the pinna.

We then compared expression of p-ERK to that of the FGF readouts *Erm* (*Etv5*) and *Pea3* (*Etv4*). Both ETS transcription factors are transcriptionally induced by FGF signaling but can have different expression domains during development, with *Erm* less restricted compared to *Pea3* in the zebrafish (Raible and Brand, 2001). Both *Erm* and *Pea3* colocalized with p-ERK in the outer regions of the pinna in the epithelium and mesenchyme (Figure 4D,E, asterisk). However, *Erm* and *Pea3* were also expressed strongly in the inner regions of the pinna (Figures 4D,E, arrowhead), in contrast with the lack of p-ERK staining in this region (Figure 4B, arrowhead), suggesting different molecular effectors operate between the outer and inner regions of the pinna. In addition, *Erm* and *Pea3* were expressed in the pinna muscle (Figures 4D,E) and *Erm* was expressed in the forming cartilage (Figure 4D). Loss of *Fgf10* in the mutant led to a reduction of *Erm* and *Pea3* expression across the pinna, with a particularly strong downregulation in the outer epithelium and mesenchyme, similar to that observed for p-ERK (Figures 4I,J, asterisk). A robust loss of *Erm* expression in the mutants was also observed at E15.5 (Supplementary Figure 6), with a reduction of expression in the mesenchyme on the outer side of the pinna cartilage. At E15.5 the loss of *Erm* in the inner pinna epithelium was particularly striking.

### Fgf10 Does Not Regulate BMP Signaling

**FIGURE 5:**
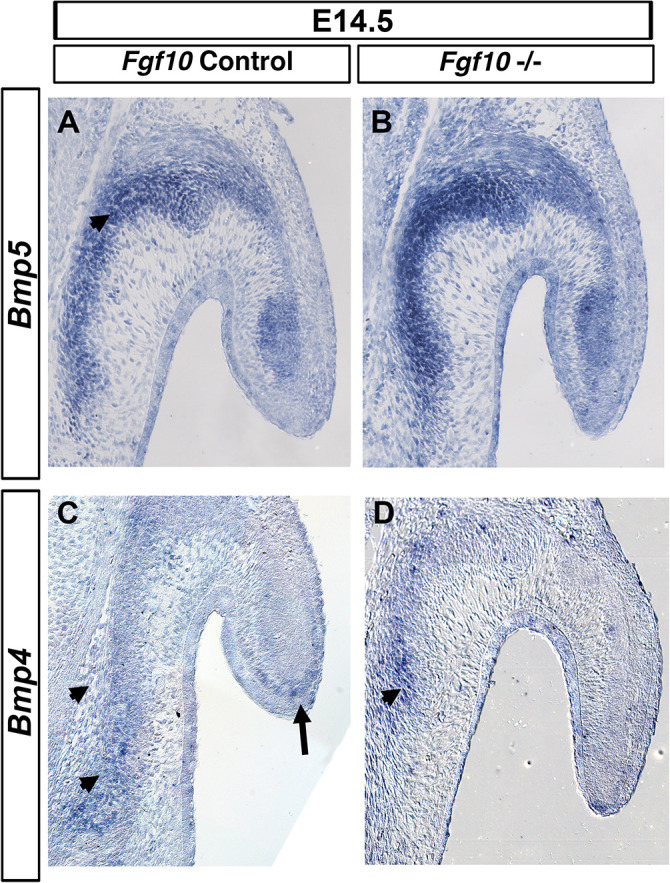
*Bmp4/5* are not regulated by Fgf10. ISH for *Bmp5* **(A,B)** and *Bmp4* **(C,D)** in control litermates **(A,C)** and *Fgf10* null mutant **(B,D)**. *Bmp5* is expressed at the base of the pinna moving into the pinna itself (arrow head in **A**). *Bmp4* is expressed in the basal cartilage (arrow heads, **C,D**) and at the very tip of the pinna (**A**, arrow). *Fgf10* null mutants maintain expression of both *Bmp5* **(B)** and *Bmp4* **(D)**.

Members of the BMP family have a role in pinna development since mutations in either *Bmp5* and *Bmp4* lead to external ear defects (King et al., 1994; Minoux et al., 2013). Mutations in *Bmp5* are responsible for the phenotype in the Short Ear mouse (King et al., 1994), while a subset of *Bmp4* hypomorphic mutant mice display a similar phenotype to the one we describe here in *Fgf10* null mutants (Minoux et al., 2013). We therefore tested for a possible genetic interaction between *Fgf10* and *Bmp4* and *5*. *Bmp5* was expressed all along the auricular cartilage (King et al., 1994), but also was observed in the adjacent auricular muscle (Figure 5A). Strong expression was maintained in the *Fgf10* mutant at E14.5 (Figure 5B). *Bmp4* is expressed at the base of the pinna and at the very tip of the extending pinna (Figure 5C; Minoux et al., 2013). These two expression domains were maintained in the *Fgf10* mutant, with robust expression at the base of the pinna (Figure 5D), in contrast to the lack of p-ERK activity in this same region (Figure 4). Despite their similar phenotype, we conclude that *Fgf10* does not sit upstream of the *Bmp4/5* pathways.

## Discussion

In this study we have shown that *Fgf10* controls pinna development by inducing proliferation of both epithelial and mesenchymal cells within the pinna, particularly at the tip, leading to extension and shaping of the pinna. Loss of *Fgf10* resulted in smaller, malformed pinnae, correlating with the dysmorphisms found in LADD syndrome, in which patients show smaller, cup-shaped ears. This characteristic malformation in patients is therefore likely to be explained by defects in proliferation. A central role of proliferation in shaping the ear is supported by our novel culture system of the whole pinna, where experimental inhibition led to an arrest of bending and elongation of the pinna at early stages of development.

One feature of murine pinna morphogenesis is the bending of the initial outgrowth toward the prospective tragus, leading to coverage of the ear canal. This rostral movement appears distinct to mice and rats and may play a role in protecting the ear canal during its development. In culture inhibition of proliferation impairs bending, in addition to the elongation of the tip. This suggests that proliferation, rather than morphogenetic movements, such as convergent extension, drive pinna morphogenesis. Higher proliferation on the outer part of the pinna combined with lower proliferation in the inner part of the pinna would be predicted to lead to an inwards bending. However, we did not detect such differences in proliferation with our BrdU labeling, but it might have been masked due to the high incorporation index of BrdU in the ear, as expected from a growing embryonic structure. Alternatively, a difference may only be evident at earlier stages. As *Fgf10* mutant mice had normal development up to E14.5, the control of proliferation at earlier stages must be provided by other signaling factors.

Analysis of Fgf10 expression using a LacZ reporter highlighted expression in both the forming pinna muscle and a subset of neural crest derived cartilage cells. Ablation of *Fgf10* specifically in the neural crest did not affect pinna development, in comparison with the null mutant. This suggests that mesodermally derived *Fgf10* is sufficient for pinna elongation. In this case the mesodermally derived Fgf10 appears to be able to compensate for loss of Fgf10 in the forming cartilage. Conditional knockout of *Fgf10* in the pinna mesoderm would be an important next step in order to investigate whether the phenotype is driven solely by loss of Fgf10 from the mesoderm.

The FGF canonical pathway ERK is an essential transducer of FGF signaling in a variety of tissues, including salivary glands (Chatzeli et al., 2017). Our data shows that ERK 1/2 also transduces Fgf10 signaling in the pinna, with loss of p-ERK in the cells lateral to the Fgf10 expressing cartilage and muscle, and at the tip of the pinna in the mutant. Interestingly, differential p-ERK was observed between the outer and inner part of the elongating pinna at E14.5, suggesting that cells on the outer part of the pinna might be more responsive to Fgf10 signaling than those on the inner side. The expression of the FGF readouts *Erm* and *Pea3*, however, did not match the expression of p-ERK, and showed high levels of expression in the inner pinna epithelium, which was dramatically reduced in the mutant. In keeping with the wider expression of *Erm* and *Pea3*, a reduction of proliferation was identified in both the inner and outer tissue in the *Fgf10* mutants. Interestingly, *Erm* and *Pea3* had slightly different expression patterns, suggesting that *Erm* and *Pea3* respond to different thresholds or follow different kinetics using the same signal (Raible and Brand, 2001). In zebrafish, for example, *Erm* required a lower level of FGF signaling for activation than *Pea3* (Roehl and Nusslein-Volhard, 2001).

The difference in ERK 1/2 phosphorylation suggests that the pinna is compartmentalized, with both a medial-lateral and proximo-distal axis. Indeed, differential gene expression has been shown between these axis with *Prx1* expressed in the inner region of the elongating pinna and absent in the outer region, while *Bmp4* and *Hoxa2* are differentially expressed along the proximo distal axis (Minoux et al., 2013). These axes are likely to be important in directing pinna morphogenesis.

The essential role of ERK pathway in pinna development is highlighted by the ERK conditional knock out in the cranial neural crest (Newbern et al., 2008). This mutant mouse displays a severe malformation of the external ear or anotia (lack of pinna). In contrast, the *Fgf10* mutant shows a much milder pinna phenotype, suggesting that Fgf10 is not the only ligand that activates ERK1/2, particularly at earlier stages. Fgf8 induces ERK 1/2 activation and the pinna is missing or defective in *Fgf8* compound heterozygous mutants (Abu-Issa et al., 2002; Moon et al., 2006). Therefore, Fgf8 could be playing a role at earlier stages of pinna development, and may compensate for the loss of *Fgf10* at later stages. In keeping with this, the expression of FGF readouts *Erm* and *Pea3* were reduced but not abolished in the *Fgf10* mutants.

Previous studies have reported a role of Fgf10 signaling in cranial muscle differentiation (Sugii et al., 2017). *Fgf10*, *Erm*, and *Pea3* were expressed in the pinna muscle, suggesting a potential role for Fgf10 in the differentiation of these tissues. In keeping with this, Fgf10 has been shown to act downstream of *Tbx1*, with loss of *Tbx1* leading to muscle defects (Kelly et al., 2004). However, the differentiated muscle marker 12–101 was unaffected in the *Fgf10* mutant pinna, indicating that muscle was able to differentiate as normal in the absence of *Fgf10*. Therefore, either Fgf10 is not involved in auricular muscle differentiation or other FGFs present in the pinna can compensate for its loss.

Others growth factors involved in cartilage differentiation and pinna development are members of the BMP family. A subset of *Bmp4* hypomorph mutant show a similar phenotype to the *Fgf10* mutant phenotype we describe here, with the pinna failing to elongate, leaving the ear canal exposed (Minoux et al., 2013). *Bmp5* mutants display small and microtic ears (King et al., 1994). Both genes are expressed in the cartilaginous condensations of the pinna (Minoux et al., 2013) overlapping with *Erm/Pea3*. In the lung epithelium, *Fgf10* regulates *Bmp4* expression (Abler et al., 2009). In the pinna of the *Fgf10* mutant, however, *Bmp4/5* expression was still present, suggesting that Fgf10 does not sit upstream of *Bmp4/5* during pinna development. It is possible, however, that FGF signaling is regulated by Bmp signaling, thus an analysis of Fgf10 expression in the pinna of *Bmp4* and *5* mutants would be an interesting next step.

Fgf10 in the pinna is likely to act through Fgfr2b given the pinna defect in patients with mutations in both parts of the pathway (Milunsky et al., 2006; Rohmann et al., 2006). The expression of *Fgfr2b* has not been followed in the pinna but this receptor is strongly expressed in cranial epithelium, with weaker mesenchymal expression described in areas such as the forming palate (Rice et al., 2004). The potential expression in both epithelium and mesenchyme suggests that Fgf10 could directly signal to both epithelial and mesenchymal tissue. The pinna has not been studied in the *Fgfr2b* knockout (De Moerlooze et al., 2000), but a similar defect might be predicted given the overlap in phenotypes between the *Fgf10* and *Fgfr2b* mouse mutants. Interestingly a pinna defect has been noted in mice with a missense mutation in *Fgfr1*, known as hush puppy (Calvert et al., 2011). Fgfr1b can act as a receptor for Fgf10, in addition to Fgfr2b (Watson and Francavilla, 2018). Hush puppy heterozygotes have small misshapen and low set ears similar to LADD syndrome patients (Calvert et al., 2011). *Fgfr1* homozygous hypomorphs also have a very reduced pinna (Trokovic et al., 2003). It is, therefore, possible that Fgf10 acts through both Fgfr2b and Fgfr1b during pinna development.

## Conclusion

In conclusion, our data provide a novel insight in the molecular mechanisms underpinning microtia in LADD syndrome and provides the base for future studies in microtia research.

## Data Availability Statement

This research did not produce any datasets and all the data is presented in the article. Material is available on request for interested researchers.

## Ethics Statement

All experiments were conducted in accordance with UK and local institutional regulations governing work with transgenic animals. All animals were culled using an approved schedule one method.

## Author Contributions

AT and JF conceived the experiments. JF and YZ conducted the experiments and undertook data analysis. MH isolated and supplied tissues from Fgf10lacZ mice. AT, JF, and YZ wrote the manuscript. All authors read drafts and added to the manuscript.

## Conflict of Interest

The authors declare that the research was conducted in the absence of any commercial or financial relationships that could be construed as a potential conflict of interest.

## Supplementary Material

Supplementary Figure 1 Counting method for serial sections of pinna. To quantify the numbers of proliferating cells a fixed region was initially defined at the tip of the extending pinna at E14.5 and E15.5. **(A)** A dotted line was drawn at the boundary between the epithelium and mesenchymal layers of the pinna. Twenty mesenchymal cells outlined by DAPI were then counted in an arc along this dotted line, with 10 cells on each side of the arc (blue dots highlight nuclei along the dotted line). The limit of the area was then further defined by addition of lines around and through the tip. **(B)** BrdU positive mesenchymal cells within the yellow domain, and BrdU positive epithelial cells within the pink domain were then counted. To quantify the inner and outer sides of the pinna. We selected an area 5 cells away from the tip region previously counted. An area the height of 10 cells was then selected and the epithelial and mesenchmal cells within that area counted.

Supplementary Figure 2 Pinna cartilage is formed by neural crest derived mesenchyme. **(A–F)** E15.5 pinna. Neural crest derived tissue in red. **(B,F)** The pinna epithelium is not labeled by Wnt1creTom and expresses Sox9 (green) **(B,D,F)** Sox9 expression in green. 12–101 muscle expression in blue. Boxed areas in **(B)** shown in **(C–F)**. Sox9 is expressed in the developing cartilage and pinna epithelium. **(C,D)** The Sox9 cells within the pinna overlap with the neural crest marker in the region of the forming cartilage. **(E,F)** The forming muscle does not overlap with the neural crest marker, in contrast to the mesenchymal Sox9.

Supplementary Figure 3 Cleft palate in *Wnt1creFgf10flfl* embryos. **(A)** Cre negative *Fgf10 flfl* control mouse showing a closed palate at E18.5. **(B)** *Wnt1creFgf10flfl* mutant mouse showing failure of palate formation at E18.5.

Supplementary Figure 4 Reduced proliferation throughout the pinna in *Fgf10* mutants. **(A)** Pinna at E15.5 showing BrdU positive cells. Boxed areas on either side of the pinna indicate the region counted. **(B)** Graphs comparing number of proliferating cells. E14.5 inner epithelium *P* = 0.011. E14.5 outer epithelium *P* = 0.0361. E14.5 inner mesenchyme *P* = 0.1578. E14.5 outer mesenchyme *P* = 0.01. E15.5 inner epithelium *P* = 0.0675. E15.5 outer epithelium *P* = 0.0169. E15.5 inner mesenchyme *P* = 0.0154. E15.5 outer mesenchyme *P* = 0.0015. *P* < 0.05 (^∗^), *P* < 0.01 (^∗∗^).

Supplementary Figure 5 *Fgf10* loss of function does not lead to an increase in cell death. IF for cleaved caspase 3 at E14.5 **(A–D)** and E15.5 **(E–H)** in control littermates **(A,B,E,F)** and *Fgf10* null mutant **(C,D,G,H)**. There are no apoptotic cells in the pinna at both stages. However, as a positive control, positive cells were evident within the ear canal at E15.5 as previously reported (**F**, arrow). Lack of *Fgf10* signaling does not increase apoptosis at the tip of the pinna **(A′,C′,E′,G′)**. The green dots are autofluorescence cells.

Supplementary Figure 6 Downregulation of *Erm* at E15.5 in *Fgf10* mutants. **(A,B)** *Erm in situ* hybridization at E15.5. **(A)** Fgf10 control littermate, **(B)** *Fgf10* mutant.
