# Supplementary figures and images for: An Essential Requirement for Fgf10 in Pinna Extension Sheds Light on Auricle Defects in LADD Syndrome
Source: Front Cell Dev Biol. 2020 Dec 10;8:609643. doi: 10.3389/fcell.2020.609643 (PMC7758485; doi:10.3389/fcell.2020.609643)

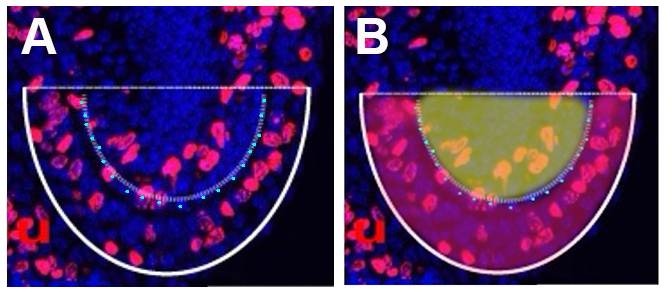

Supplement: Supplementary Figure 1 — Counting method for serial sections of pinna. To quantify the numbers of proliferating cells a fixed region was initially defined at the tip of the extending pinna at E14.5 and E15.5. (A) A dotted line was drawn at the boundary between the epithelium and mesenchymal layers of the pinna. Twenty mesenchymal cells outlined by DAPI were then counted in an arc along this dotted line, with 10 cells on each side of the arc (blue dots highlight nuclei along the dotted line). The limit of the area was then further defined by addition of lines around and through the tip. (B) BrdU positive mesenchymal cells within the yellow domain, and BrdU positive epithelial cells within the pink domain were then counted. To quantify the inner and outer sides of the pinna. We selected an area 5 cells away from the tip region previously counted. An area the height of 10 cells was then selected and the epithelial and mesenchmal cells within that area counted. [file Image_1.JPEG]

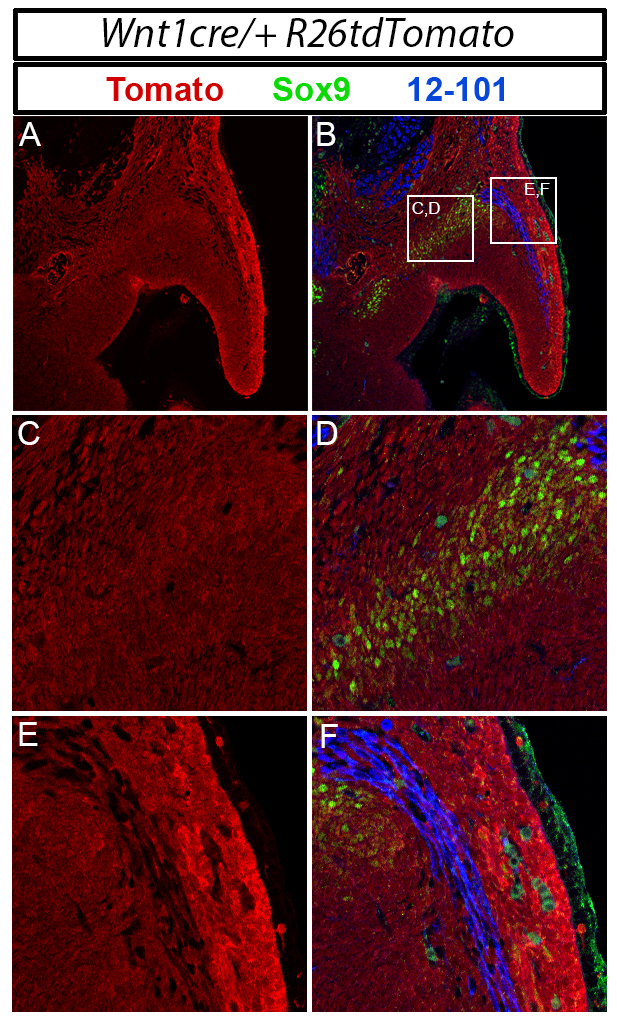

Supplement: Supplementary Figure 2 — Pinna cartilage is formed by neural crest derived mesenchyme. (A–F) E15.5 pinna. Neural crest derived tissue in red. (B,F) The pinna epithelium is not labeled by Wnt1creTom and expresses Sox9 (green) (B,D,F) Sox9 expression in green. 12–101 muscle expression in blue. Boxed areas in (B) shown in (C–F). Sox9 is expressed in the developing cartilage and pinna epithelium. (C,D) The Sox9 cells within the pinna overlap with the neural crest marker in the region of the forming cartilage. (E,F) The forming muscle does not overlap with the neural crest marker, in contrast to the mesenchymal Sox9. [file Image_2.TIF]

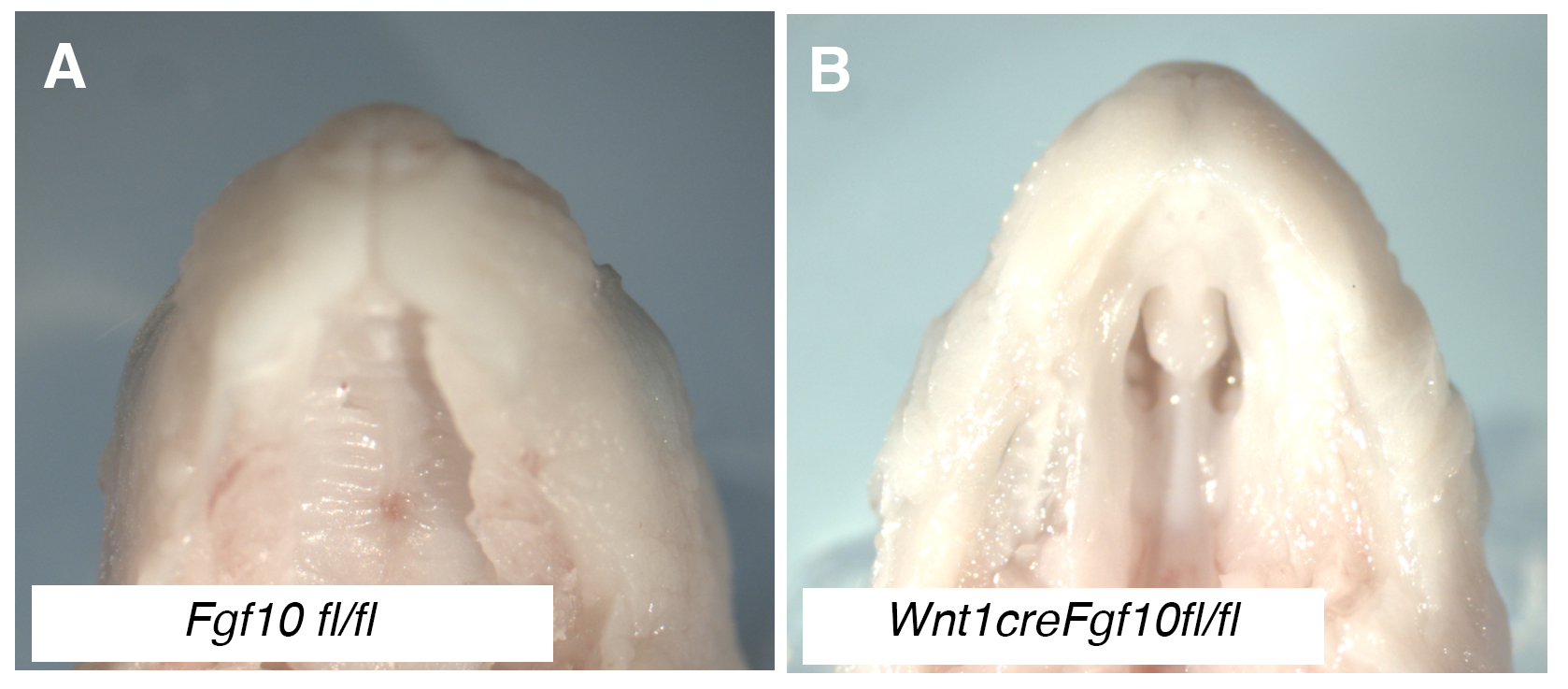

Supplement: Supplementary Figure 3 — Cleft palate in Wnt1creFgf10flfl embryos. (A) Cre negative Fgf10 flfl control mouse showing a closed palate at E18.5. (B) Wnt1creFgf10flfl mutant mouse showing failure of palate formation at E18.5. [file Image_3.TIF]

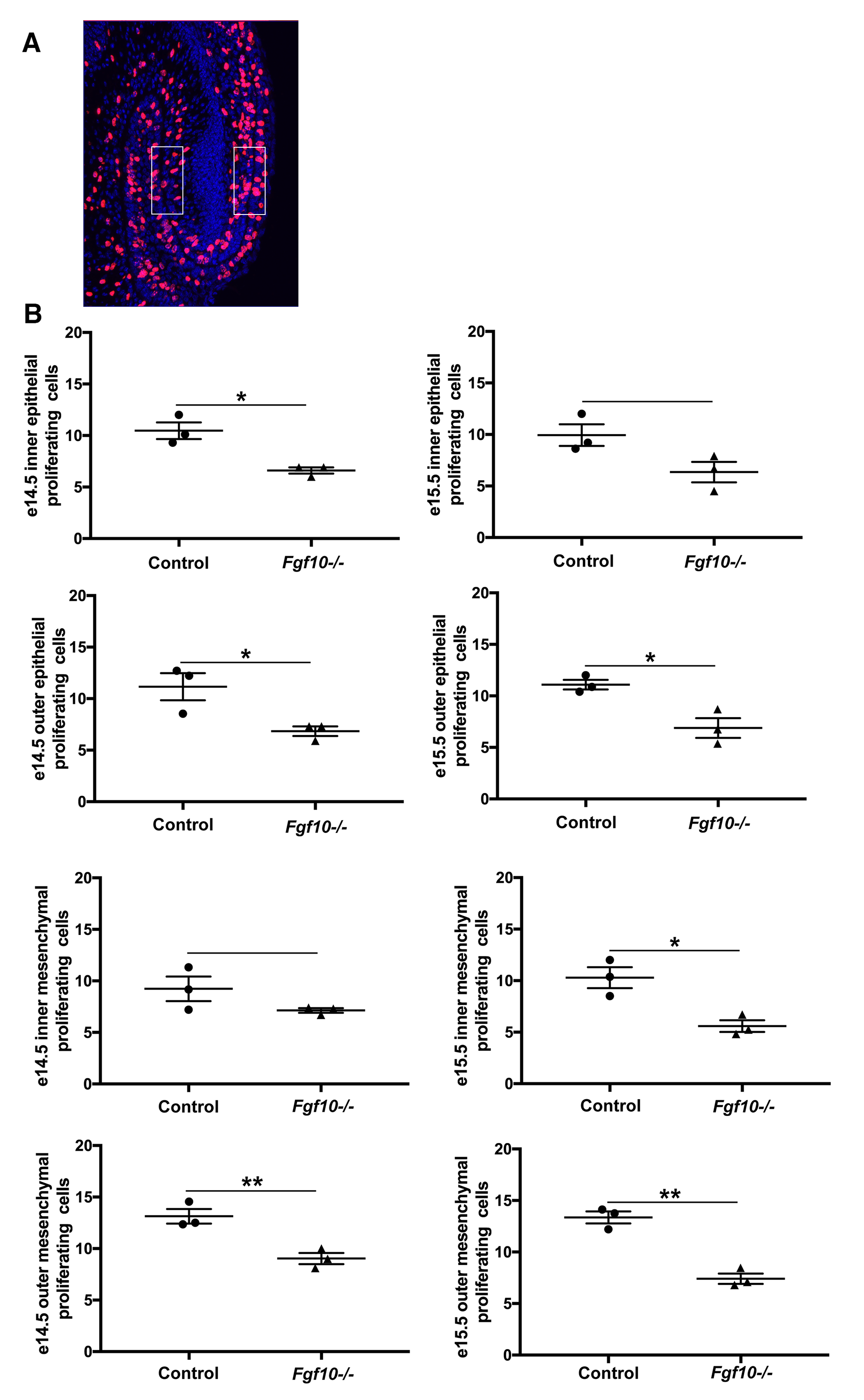

Supplement: Supplementary Figure 4 — Reduced proliferation throughout the pinna in Fgf10 mutants. (A) Pinna at E15.5 showing BrdU positive cells. Boxed areas on either side of the pinna indicate the region counted. (B) Graphs comparing number of proliferating cells. E14.5 inner epithelium P = 0.011. E14.5 outer epithelium P = 0.0361. E14.5 inner mesenchyme P = 0.1578. E14.5 outer mesenchyme P = 0.01. E15.5 inner epithelium P = 0.0675. E15.5 outer epithelium P = 0.0169. E15.5 inner mesenchyme P = 0.0154. E15.5 outer mesenchyme P = 0.0015. P < 0.05 (∗), P < 0.01 (∗∗). [file Image_4.TIF]

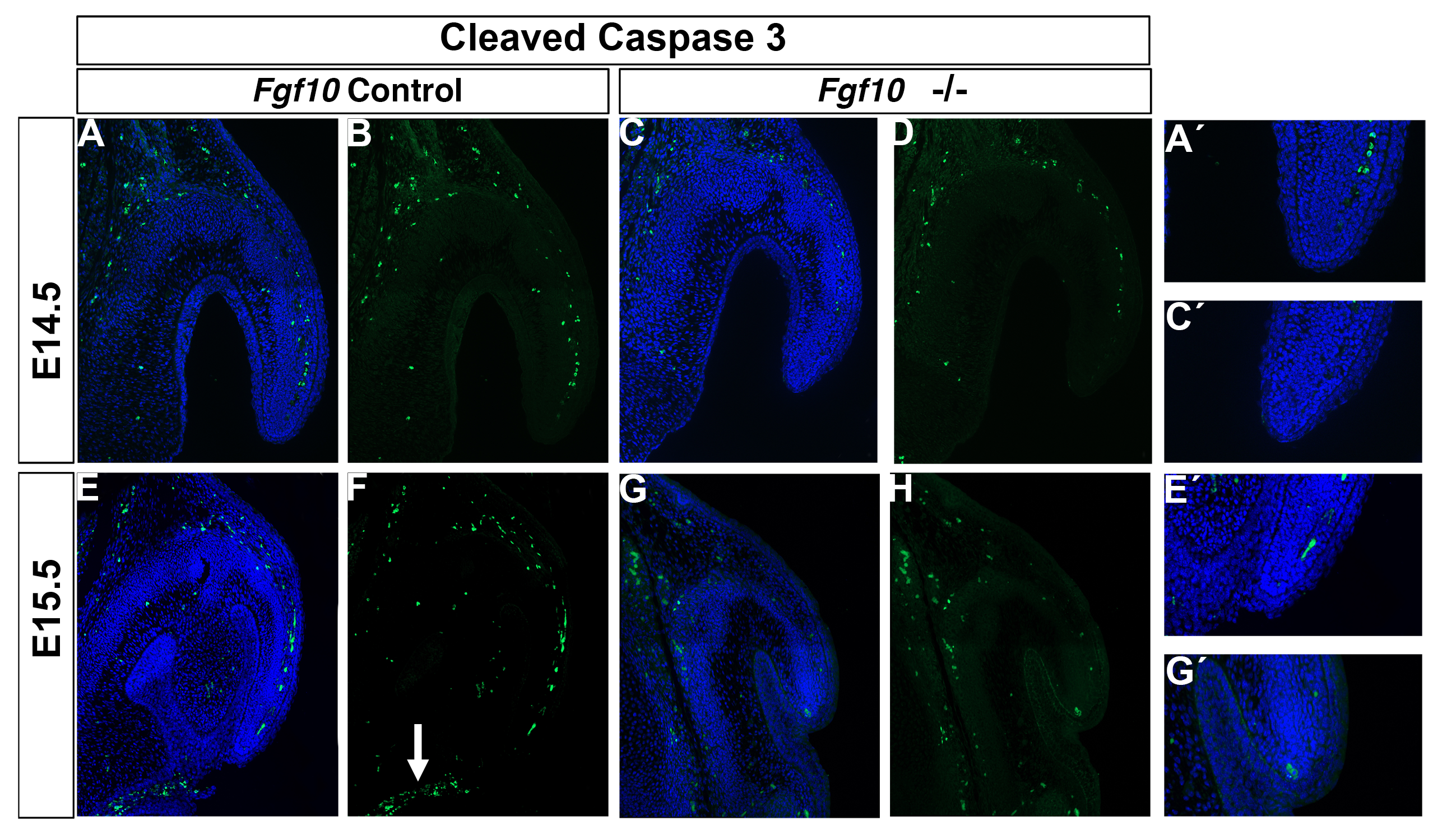

Supplement: Supplementary Figure 5 — Fgf10 loss of function does not lead to an increase in cell death. IF for cleaved caspase 3 at E14.5 (A–D) and E15.5 (E–H) in control littermates (A,B,E,F) and Fgf10 null mutant (C,D,G,H). There are no apoptotic cells in the pinna at both stages. However, as a positive control, positive cells were evident within the ear canal at E15.5 as previously reported (F, arrow). Lack of Fgf10 signaling does not increase apoptosis at the tip of the pinna (A′,C′,E′,G′). The green dots are autofluorescence cells. [file Image_5.TIF]

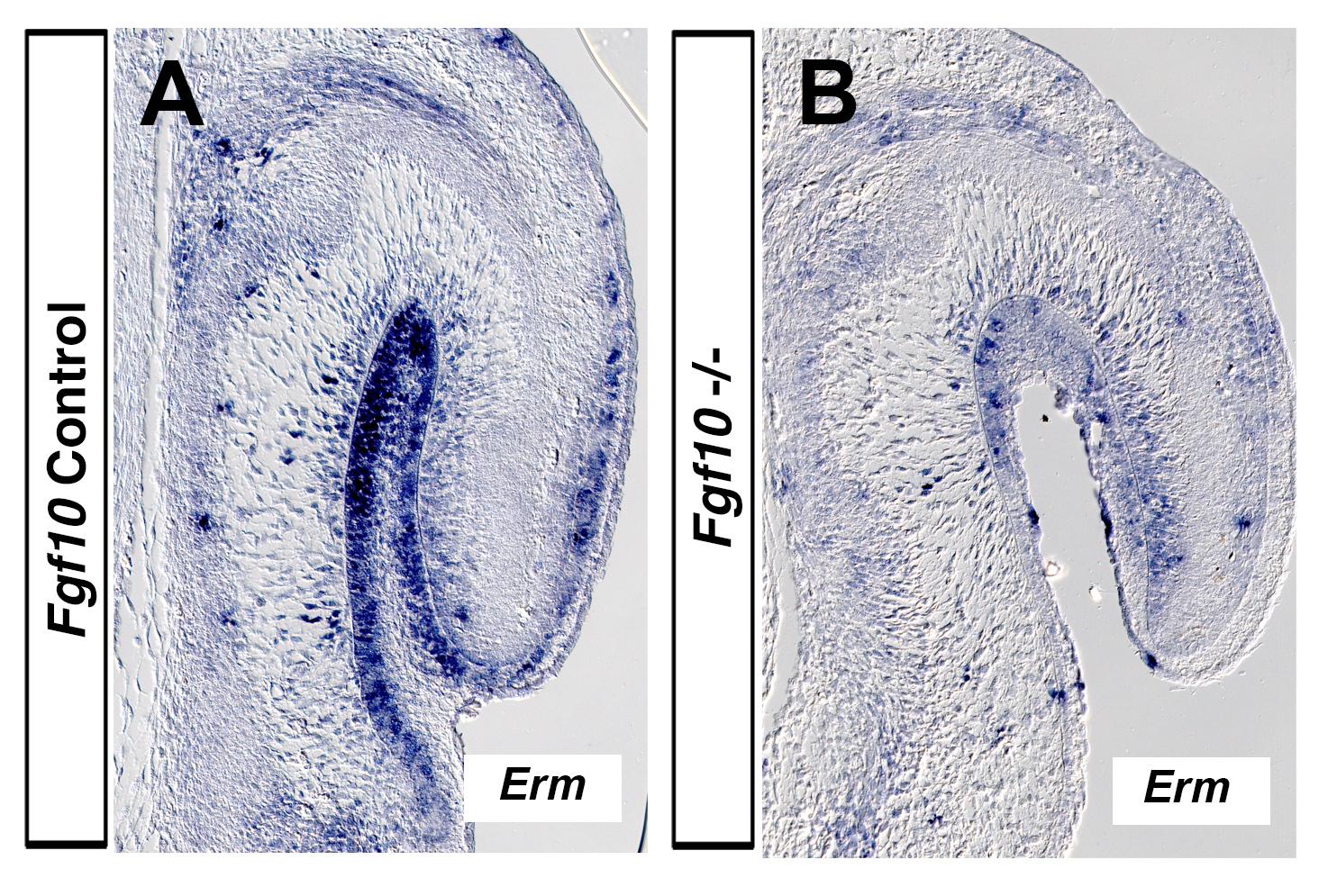

Supplement: Supplementary Figure 6 — Downregulation of Erm at E15.5 in Fgf10 mutants. (A,B) Erm in situ hybridization at E15.5. (A) Fgf10 control littermate, (B) Fgf10 mutant. [file Image_6.TIF]
